# Neural pathways from hypothalamic orexin neurons to the ventrolateral preoptic area mediate sleep impairments induced by conditioned fear

**DOI:** 10.3389/fnins.2023.1122803

**Published:** 2023-03-14

**Authors:** Caifen Ma, Ning Zhou, Kang Ma, Jiandong Niu, Ting Mi, Zhenquan He, Yujun Wen, Chunhong Liu, Zhongyi He, Jianguo Niu

**Affiliations:** ^1^Department of Human Anatomy, School of Basic Medical Sciences, Ningxia Medical University, Yinchuan, China; ^2^Key Laboratory of Craniocerebral Diseases of Ningxia Hui Autonomous Region, Ningxia Medical University, Yinchuan, China; ^3^Department of Neurology, General Hospital of Ningxia Medical University, Yinchuan, China

**Keywords:** conditioned fear, sleep impairments, orexin, VLPO, neural pathways

## Abstract

**Introduction:**

Fear and sleep impairments common co-exist, but the underlying mechanisms remain unclear. Hypothalamic orexinergic neurons are involved in the regulation of sleep-wake and fear expression. The ventrolateral preoptic area (VLPO) is an essential brain region to promote sleep, and orexinergic axonal fibers projecting to the VLPO are involved in the maintenance of sleep-wake. Neural pathways from hypothalamic orexin neurons to the VLPO might mediate sleep impairments induced by conditioned fear.

**Methods:**

To verify above hypothesis, electroencephalogram (EEG) and electromyogram (EMG) were recorded for analysis of sleep-wake states before and 24 h after conditioned fear training. The retrograde tracing technique and immunofluorescence staining was used to identify the projections from the hypothalamic orexin neurons to the VLPO and to observe their activation in mice with conditioned fear. Moreover, optogenetic activation or inhibition of hypothalamic orexin-VLPO pathways was performed to observe whether the sleep-wake can be regulated in mice with conditioned fear. Finally, orexin-A and orexin receptor antagonist was administered into the VLPO to certify the function of hypothalamic orexin-VLPO pathways on mediating sleep impairments induced by conditioned fear.

**Results:**

It was found that there was a significant decrease in the non-rapid eye movement (NREM) and rapid eye movement (REM) sleep time and a significant increase in the wakefulness time in mice with conditioned fear. The results of retrograde tracing technique and immunofluorescence staining showed that hypothalamic orexin neurons projected to the VLPO and observed the CTB labeled orexin neurons were significantly activated (c-Fos+) in the hypothalamus in mice with conditioned fear. Optogenetic activation of hypothalamic orexin to the VLPO neural pathways significantly decreased NREM and REM sleep time and increased wakefulness time in mice with conditioned fear. A significant decrease in NREM and REM sleep time and an increase in wakefulness time were observed after the injection of orexin-A into the VLPO, and the effects of orexin-A in the VLPO were blocked by a pre-administrated dual orexin antagonist (DORA).

**Conclusion:**

These findings suggest that the neural pathways from hypothalamic orexinergic neurons to the VLPO mediate sleep impairments induced by conditioned fear.

## Introduction

Fear is one of the primitive emotions shared by human and animals, which can stimulate a series of defense behavior to make the body adapt to the environment, survivor and development (Kaplan et al., 2022). In recent years, an increased number of researches have shown that amygdala, hippocampus, hypothalamus, locus coeruleus and medial prefrontal cortex are the main nucleus involved in regulating fear expression (Burwell et al., 2004; Do-Monte et al., 2015; Soya et al., 2017). Fear often causes sleep impairments, such as insomnia, frequent nightmares, and sleep disruption (Pace-Schott et al., 2022). The animal models of conditioned fear have been widely used to study fear-related diseases (Soya et al., 2013). At present, quantitative electroencephalogram (EEG), functional magnetic resonance imaging (fMRI), positron emission tomography (PET) and other technologies have been used to study the mechanism of fear-related sleep impairments (Zhang et al., 2019; Zhou et al., 2021; Chellappa and Aeschbach, 2022). However, current researches cannot fully explain its mechanisms, and many researchers believe that it may be a new breakthrough to study its mechanisms at the level of neural circuits (Baek et al., 2019).

Orexins also known as the hypocretins (Hcrts), are two peptides derived from a single precursor produced in the posterior lateral hypothalamus. The orexin system has been associated with numerous physiological functions, including sleep/arousal, attention, stress response, and fear memory expression (Sakurai, 2007; Li et al., 2014; Mahler et al., 2014; Sakurai, 2014; Flores et al., 2015). In recent years, optogenetic and pharmacogenetic researches have demonstrated the causal relationship between the activation of orexin neurons and arousal nodes in other brain regions (Adamantidis et al., 2007; Tsunematsu et al., 2013), for example, the stimulation of orexin neurons directly activates noradrenergic neurons in the locus coeruleus, increasing arousal (Carter et al., 2012). Although orexin neurons have been shown to regulate the expression of fear through important regions such as the locus coeruleus and amygdala (Steiner et al., 2012; Soya et al., 2017), it is still unclear how orexin neurons regulate sleep impairments induced by conditioned fear.

Located in the anterior part of hypothalamus, the ventrolateral preoptic area (VLPO) is an essential brain region to promote sleep (Sherin et al., 1996; Szymusiak et al., 1998). The VLPO contains plentiful sleep-promoting neurons, such as GABAergic and galaninergic neurons (Venner et al., 2016; Herrera et al., 2016). The lesions of VLPO decrease time of rapid eye movement (REM) and non-rapid eye movement (NREM) sleep (Lu et al., 2000). Optogenetic specific activation of GABAergic or galaninergic neurons in the VLPO promotes sleep and reduces wakefulness (Kroeger et al., 2018). VLPO neurons have reciprocal connections with orexin neurons of hypothalamus (Peyron et al., 1998). GABAergic neurons in the VLPO are inhibited by orexin neurons during wakefulness (De Luca et al., 2022). Based on these studies, it is quite probable that the hypothalamic orexin-VLPO neural pathways mediate sleep impairments induced by conditioned fear, but no relevant studies have been reported so far. To verify above hypotheses, EEG and electromyogram (EMG) were recorded for analysis of sleep-wake states before and 24 h after conditioned fear training. The retrograde tracing technique and immunofluorescence staining were used to identify the pathways from the hypothalamic orexin neurons to the VLPO and to observe their activation in mice with conditioned fear.

## Results

### Sleep impairments occurred in the mice after conditioned fear training

**FIGURE 1:**
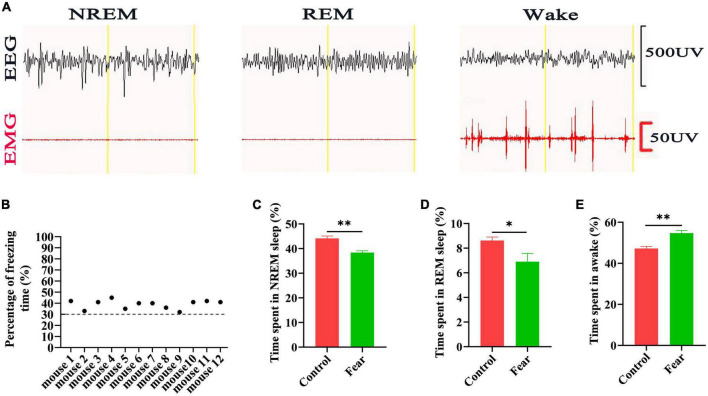
Sleep impairments occurred in the mice after conditioned fear training. **(A)** An example of EEG and EMG recording traces during non-rapid eye movement (NREM) sleep, rapid eye movement (REM) sleep and wakefulness in mice. **(B)** The percentage of freezing time in mice with conditioned fear (*n* = 12 mice). **(C)** The comparison of the percentage of 24 h NREM sleep time in control and conditioned fear mice (*n* = 5 mice per group). **(D)** The comparison of the percentage of 24 h REM sleep time in control and conditioned fear mice (*n* = 5 mice for per group). **(E)** The comparison of the percentage of 24 h wakefulness time in control and conditioned fear mice (*n* = 5 mice per group). *P < 0.05, ^**^P < 0.01. EEG, electroencephalogram; EMG, electromyogram.

The freezing behavior of the mice (*n* = 12) was tested after the conditioned fear training, and it was found that the percentage of freezing time of each mouse reached 30% (Figure 1B). Sleep-wakefulness analysis was performed based on the 24 h EEG and EMG signals collected (Figure 1A). The sleep-wakefulness analysis revealed a significant decrease in NREM and REM sleep time (control vs. fear group: 44.14 ± 2.17% vs. 38.4 ± 1.62% and 8.62 ± 0.64% vs. 6.9 ± 1.4%, *t* = 4.743 and *t* = 2.368, *P* = 0.002 and *P* = 0.045, *n* = 5 mice per group) (Figures 1C, D), and a significant increase in wakefulness time (control vs. fear group: 47.24 ± 2.08% vs. 54.7 ± 2.8%, *t* = 4.782, *P* = 0.001, *n* = 5 mice per group) (Figure 1E) 24 h after conditioned fear training.

### Hypothalamic orexin neurons projected to the VLPO

**FIGURE 2:**
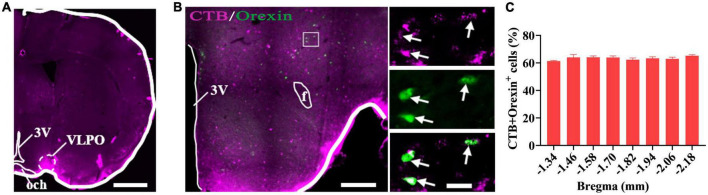
Hypothalamic orexin neurons projected to the VLPO. **(A)** The image of injection site of Cholera Toxin B Subunit (CTB) in the VLPO in mice. **(B)** The images of CTB (pink) and orexin (green) immunofluorescence staining in the hypothalamus, with squared areas enlarged in the right images. **(C)** Quantification of the percentage of CTB^+^ and orexin^+^ colocalization in all orexin^+^ neurons in each section of hypothalamus (*n* = 3 mice). Scar bars: **(A)** 1,000 μm, **(B)** left image 500 μm, right images 50 μm. 3V, 3rd ventricle; och, optic chiasm; VLPO, ventrolateral preoptic nucleus; f, fornix.

After injections of Cholera Toxin B Subunit (CTB) as retrograde tracer into the VLPO of wild type (WT) mice (Figure 2A), and immunofluorescence staining for CTB and orexin neurons in the coronal sections containing hypothalamus of mice, a large number of CTB labeled cell bodies and orexin^+^ neurons were observed in hypothalamus (Figure 2B), and it was found that 63.41 ± 1.96% of the CTB labeled cell bodies were immunopositive for orexin neurons (Figure 2C). This result indicates that the hypothalamic orexin neurons projected to the VLPO.

### Hypothalamic orexin-VLPO neural pathways were activated by conditioned fear

**FIGURE 3:**
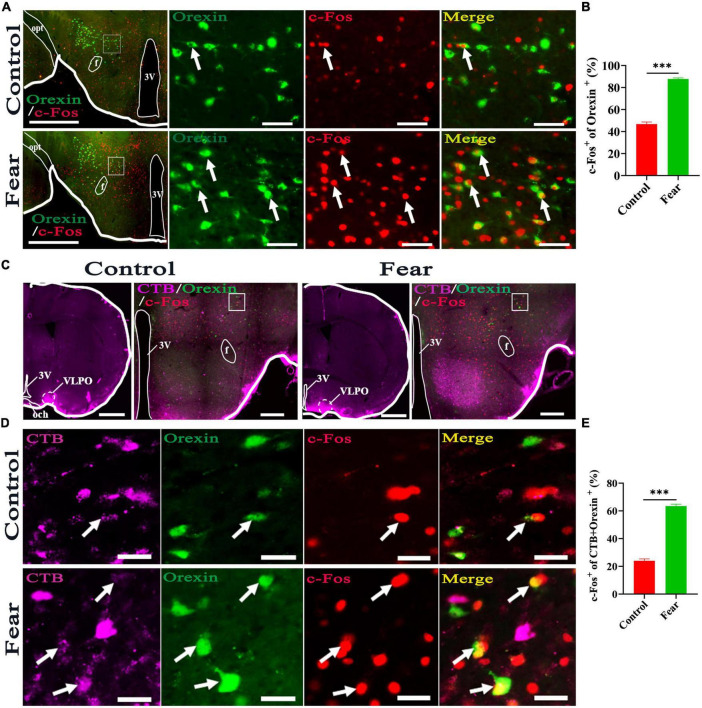
Hypothalamic orexin-VLPO neural pathways were activated by conditioned fear. **(A)** The images of immunofluorescence staining for orexin (green) and c-Fos (red) in the hypothalamus of control and conditioned fear mice, with squared areas enlarged in the right images. **(B)** The comparison of the percentage of orexin neurons that were activated (c-Fos^+^) in control and conditioned fear mice (*n* = 4 mice per group). **(C)** The image injection site of Cholera Toxin B Subunit (CTB) in the VLPO and the images of CTB (pink), orexin (green) and c-Fos (red) immunofluorescence staining of the hypothalamus in control and conditioned fear mice. **(D)** The enlarged image of CTB, orexin, c-Fos and their merge in control and conditioned fear mice. **(E)** The comparison of the percentage of CTB-labeled orexin neurons that were activated (c-Fos^+^) in control and conditioned fear mice (*n* = 4 mice per group). Scar bars: **(A)** left images 500 μm, right images 100 μm, **(C)** left image 1,000 μm, right image 500 μm, **(D)** 100 μm. 3V, 3rd ventricle; opt, optic tract; f, fornix; och, optic chiasm; VLPO, ventrolateral preoptic nucleus. ^***^P < 0.001.

Immunofluorescence staining for orexin and c-Fos was conducted in the coronal sections containing hypothalamus. The orexin^+^ neurons and c-Fos^+^ cells were observed in hypothalamus (Figure 3A), and it was found that some of the orexin^+^ neurons were also c-Fos^+^. Counting results showed that the percentage of double-stained neurons were significantly increased in mice with conditioned fear (control vs. fear group: 46.83 ± 3.84% vs. 87.83 ± 1.91%, *t* = 19.13, *P* = 0.0001, *n* = 4 mice per group) (Figure 3B). In the mice with CTB injections into the VLPO (Figure 3C) and conditioned fear trainings, immunofluorescence staining for CTB, c-Fos and orexin neurons were conducted in the coronal sections containing hypothalamus, and the CTB labeled cell bodies, c-Fos^+^ cells and orexin^+^ neurons were observed in hypothalamus (Figures 3C, D). Counting results showed that the percentage of triple-stained neurons in the conditioned fear mice were significantly higher than that in the control mice (control vs. fear group: 24.03 ± 2.66% vs. 63.5 ± 2.72%, *t* = 20.76, *P* = 0.0001, *n* = 4 mice per group) (Figure 3E). These results demonstrated that the hypothalamic orexin-VLPO neural pathways were activated by conditioned fear.

### Hypothalamic orexin neurons involved in regulating sleep-wake in mice with conditioned fear

**FIGURE 4:**
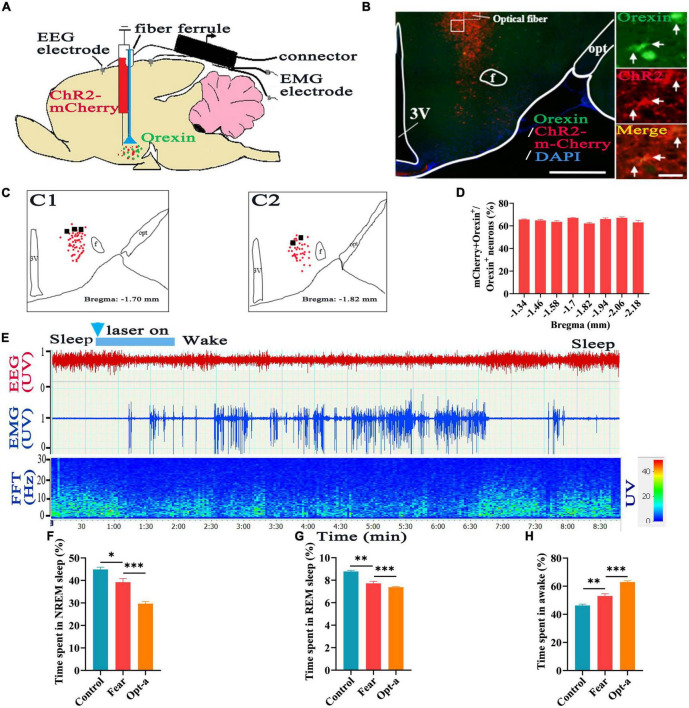
Hypothalamic orexin neurons involved in regulating sleep-wake in mice with conditioned fear. **(A)** Schematic configuration of hypothalamic viral injection, optical fiber implantation, EEG and EMG recordings. **(B)** The images of expression of ChR2-mCherry and implantation site of optical fiber in the hypothalamus in Hcrt-Cre mice, with squared areas enlarged in the right images. **(C)** The expression of the of ChR2-mCherry (red spot) and implantation site (black spot) of the optical fiber in the hypothalamus (*n* = 5 mice). **(D)** Quantification of the percentage of ChR2-mCherry^+^ and orexin^+^ colocalization in all orexin^+^ neurons in each section of hypothalamus (*n* = 3 mice). **(E)** An example of sleep-wake recording from mice with conditioned fear expressing ChR2-mCherry in the hypothalamus following optogenetic activation of orexin cell bodies during sleep (blue bars; 120 s, 10 Hz, 10 ms pulses). **(F)** The comparison of the percentage of 24 h non-rapid eye movement (NREM) sleep time in control, conditioned fear and optogenetic activation mice (*n* = 5 per group mice). **(G)** The comparison of the percentage of 24 h rapid eye movement (REM) sleep time in control, conditioned fear and optogenetic activation mice (*n* = 5 mice per group). **(H)** The comparison of the percentage of 24 h wakefulness time in control, conditioned fear and optogenetic activation mice (*n* = 5 mice per group). Scar bars: **(B)** left image 500 μm, right images 50 μm. *P < 0.05, ^**^P < 0.01, ^***^P < 0.001. Opt-a, optogenetic activation; 3V, 3rd ventricle; opt, optic tract; f, fornix.

To determine if the orexin neurons involved in regulating sleep-wake in mice with conditioned fear, the rAAV-EF1a-DIO-hChR2-mCherry was injected into hypothalamus of Hcrt-IRES-Cre mice (Figure 4A), and optical fiber was implanted at the injection site (Figures 4B, C), the optogenetic activation and EEG/EMG recording were conducted. After immunofluorescence staining in section containing hypothalamus, it was found that 64.9 ± 1.54% of orexin^+^ neurons were labeled with ChR2-mCherry (Figures 4B, D). EEG/EMG recording showed that there was a significant decrease in the NREM and REM sleep time and a significant increase in the wakefulness time in mice with conditioned fear compared with that in control mice (control vs. fear group: 44.9 ± 2.45% vs. 39.2 ± 3.58%, 8.78 ± 0.23% vs. 7.72 ± 0.41%, 46.32 ± 2.09% vs. 53.04 ± 3.58%, *t* = 3.003, 5.008, and 3.624, *P* = 0.017, 0.001, and 0.007, *n* = 5 mice per group) (Figures 4F, H). When optogenetic activation was applied to the orexin neurons in the hypothalamus, the EEG/EMG signatures representing wakefulness were observed instantaneously (Figure 4E). Continuous EEG/EMG recordings indicated that there were much more decreases in NREM and REM sleep time and increases in wakefulness time in mice with conditioned fear after optogenetic activation of hypothalamic orexin neurons (fear vs. opt-a group: 39.2 ± 3.58% vs. 29.68 ± 2.08%, 7.72 ± 0.41% vs. 7.28 ± 0.37%, 53.04 ± 3.58% vs. 62.94 ± 1.96%, *t* = 5.152, 4.516, and 5.407, *P* = 0.0009, 0.0001, and 0.0006, *n* = 5 mice per group) (Figures 4F, H).

### Activation of hypothalamic orexin-VLPO neural pathways changed sleep-wake in mice with conditioned fear

**FIGURE 5:**
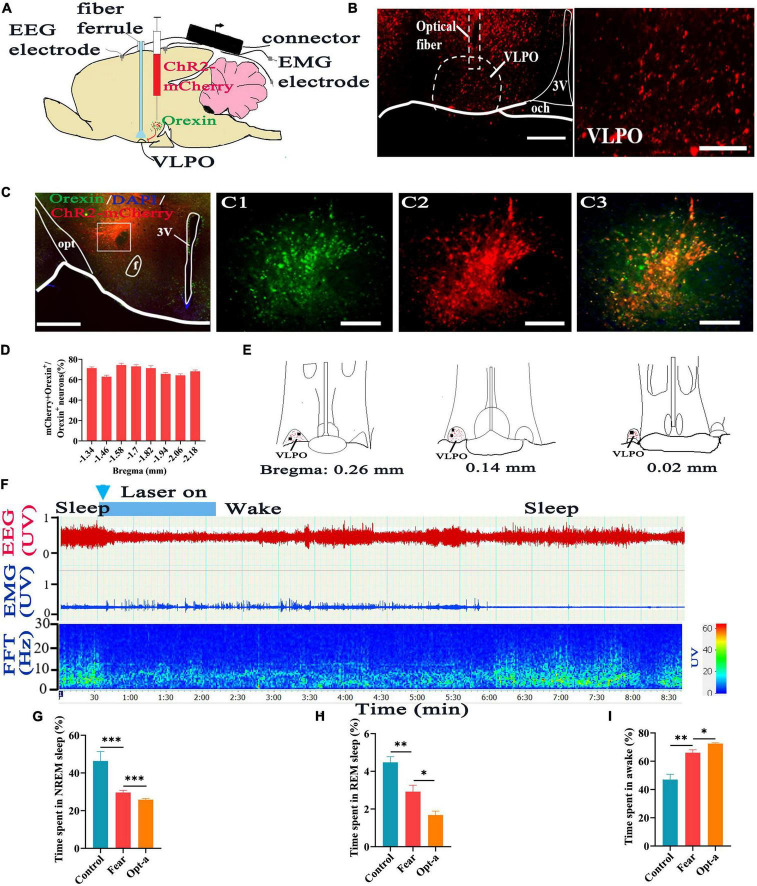
Optogenetic activation of the hypothalamic orexin-VLPO neural pathways changed sleep-wake in mice with conditioned fear. **(A)** Schematic configuration of hypothalamic viral injection, optical fiber implantation in the VLPO, EEG and EMG recordings. **(B)** The images of implantation site of optical fiber and the distribution of ChR2-mCherry terminals in the VLPO of Hcrt-Cre mice. **(C)** The images of immunofluorescence staining for ChR2-mCherry (red) and orexin (green) in the hypothalamus of Hcrt-Cre mice, with squared areas enlarged in the right images. **(D)** Quantification of the percentage of ChR2-mCherry^+^ and orexin^+^ colocalization in all orexin^+^ neurons in each section of hypothalamus (*n* = 3 mice). **(E)** The distribution of ChR2-mCherry terminals (red spot) and implantation site (black spot) of the optical fiber in the VLPO (*n* = 5 mice). **(F)** An example of sleep-wake recording from mice with conditioned fear expressing ChR2-mCherry in the hypothalamus following optogenetic activation of orexin axonal terminals in the VLPO during sleep (blue bars; 120 s, 10 Hz, 10 ms pulse). **(G)** The comparison of the percentage of 24 h non-rapid eye movement (NREM) sleep time in control, conditioned fear, and optogenetic activation mice (*n* = 5 mice per group). **(H)** The comparison of the percentage of 24 h rapid eye movement (REM) sleep time in control, conditioned fear, and optogenetic activation mice (*n* = 5 mice per group). **(I)** The comparison of the percentage of 24 h wakefulness time in control, conditioned fear, and optogenetic activation mice (*n* = 5 mice per group). Scar bars: **(B)** left image 200 μm, right image 100 μm, **(C)** left image 500 μm, right images 100 μm. *P < 0.05, ^**^P < 0.01, ^***^P < 0.001. Opt-a, optogenetic activation; 3V, 3rd ventricle; opt, optic tract; f, fornix; och, optic chiasm; VLPO, ventrolateral preoptic nucleus.

To find out whether the hypothalamic orexin-VLPO neural pathways regulate sleep-wake in mice with conditioned fear, firstly, the rAAV-EF1a-DIO-ChR2-mCherry was injected into the hypothalamus of Hcrt-IRES-Cre mice to label the orexin neurons (Figures 5A, C), and the optical fiber was implanted into the VLPO to activate the orexinergic axonal terminals in the VLPO (Figures 5B, E). By immunofluorescence staining, the specific labeling of orexin^+^ neurons by ChR2-mCherry were observed in hypothalamus, and counting results showed that the 68.9 ± 2.73% of mCherry-expressing cells overlapped with orexin^+^ neurons (Figures 5C, D). Consistent with the experiment we just showed above, the time of NREM and REM sleep was shortened and that of wakefulness time was prolonged in mice with conditioned fear (control vs. fear group: 45.99 ± 11.06% vs. 30 ± 2.06%, 4.48 ± 0.66% vs. 2.92 ± 0.77%, 47.0 ± 8.4% vs. 66.06 ± 4.52%, *t* = 4.264, 3.439, and 4.465, *P* = 0.001, 0.009, and 0.002, *n* = 5 mice per group) (Figures 5G, I). Optogenetic activation of orexinergic axonal terminals in the VLPO effectively induced the transition from sleep to wake in mice with conditioned fear (Figure 5F). The further decreases of NREM and REM sleep time and further increases of wakefulness time were observed in mice with conditioned fear after optogenetic activation of orexinergic axonal terminals in the VLPO (fear vs. opt-a group: 30 ± 2.06% vs. 25.89 ± 1.53%, 2.92 ± 0.77% vs. 1.68 ± 0.46%, 66.06 ± 4.52% vs. 72.53 ± 1.23%, *t* = 4.799, 3.092, and 2.332, *P* = 0.0002, 0.015, and 0.048, *n* = 5 mice per group) (Figures 5G, I).

**FIGURE 6:**
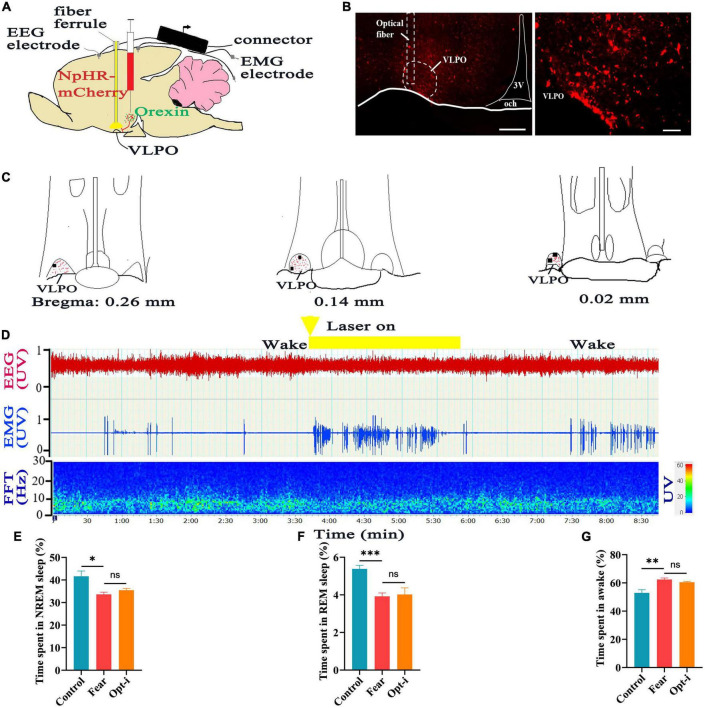
Optogenetic inhibition of the hypothalamic orexin-VLPO neural pathways did not change sleep-wake in mice with conditioned fear. **(A)** Schematic configuration of hypothalamus viral injection, optical fiber implantation in the VLPO and EEG, EMG recordings. **(B)** The images of implantation site of optical fiber and the distribution of NpHR3.0-mCherry terminals in the VLPO of Hcrt-Cre mice. **(C)** The distribution of NpHR3.0-mCherry terminals (red spot) and implantation site (black spot) in the VLPO (*n* = 5 mice). **(D)** An example of sleep-wake recording from mice with conditioned fear expressing NpHR3.0-mCherry in the hypothalamus following optogenetic inhibition of orexin axonal terminals in the VLPO during the wakefulness (yellow bars; 120 s, 10 Hz, 10 ms pulse). **(E)** The comparison of the percentage of 24 h non-rapid eye movement (NREM) sleep time in control, conditioned fear, and optogenetic inhibition mice (*n* = 5 mice per group). **(F)** The comparison of the percentage of 24 h rapid eye movement (REM) sleep time in control, conditioned fear, and optogenetic inhibition mice (*n* = 5 mice per group). **(G)** The comparison of the percentage of 24 h wakefulness time in control, conditioned fear, and optogenetic inhibition mice (*n* = 5 mice per group). Scar bars: **(B)** left image 200 μm, right image 100 μm. *P < 0.05, ^**^P < 0.01, ^***^P < 0.001. Opt-i, optogenetic inhibition; 3V, 3rd ventricle; och, optic chiasm; VLPO, ventrolateral preoptic nucleus.

Additionally, reversal experiments have been tried by injection of rAAV-EF1a-DIO-NpHR3.0-mCherry into hypothalamus of Hcrt-IRES-Cre mice to label the orexin neurons, and implantation of optical fiber into the VLPO to inhibit the local orexinergic axonal terminals (Figures 6A, C). Immunofluorescence images displayed that there were large number of orexinergic axonal fibers and terminals labeled by NpHR3.0-mCherry in the VLPO (Figure 6B). Consistent with the results showed above, same changes in sleep-wake were observed, namely, a significant decrease in the NREM and REM sleep time and a significant increase in the wakefulness time were occurred in mice with conditioned fear (control vs. fear group: 41.66 ± 5.19% vs. 33.66 ± 2.10%, 5.68 ± 0.60% vs. 3.96 ± 0.43%, 52.96 ± 4.95% vs. 62.42 ± 2.44%, *t* = 3.195, 5.283, and 3.833, *P* = 0.013, 0.0007, and 0.005, *n* = 5 mice per group) (Figures 6E, G). It wasn’t as preconceived, optical simulation of NpHR3.0-mCherry-labeled axonal terminals in the VLPO did not induce an immediate wake-to-sleep transition in mice with conditioned fear (Figure 6D), and did not cause a significant change in the time of sleep and wakefulness (fear vs. opt-i group: 33.66 ± 2.10% vs. 35.48 ± 1.75%, 3.96 ± 0.43% vs. 4.02 ± 0.79%, 62.42 ± 2.44% vs. 60.5 ± 1.28%, *t* = 1.488, 0.251, and 1.560, *P* = 0.175, 0.808, and 0.157, *n* = 5 mice for each group) (Figures 6E, G).

### Orexin-A in the VLPO increased wakefulness time and decreased sleep time in mice with conditioned fear

**FIGURE 7:**
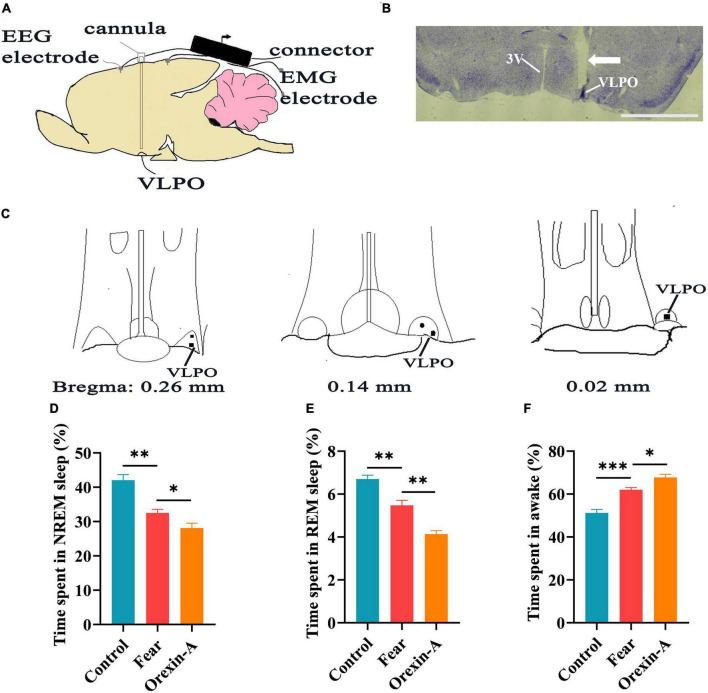
The injection of orexin-A into the VLPO changed sleep-wake in mice with conditioned fear. **(A)** Schematic of cannula implantation into the VLPO and EEG, EMG recordings. **(B)** The image of implantation site of cannula in the VLPO of mice. **(C)** The black spot representing the placement of the cannula in the VLPO of mice (*n* = 5 mice). **(D)** The comparison of the percentage of 24 h non-rapid eye movement (NREM) sleep time in control, conditioned fear, and orexin-A mice (*n* = 5 mice per group). **(E)** The comparison of the percentage of 24 h rapid eye movement (REM) sleep time in control, conditioned fear and orexin-A mice (*n* = 5 mice per group). **(F)** The comparison of the percentage of 24 h wakefulness time in control, conditioned fear and orexin-A mice (*n* = 5 mice per group). **(B)** 1,000 μm. *P < 0.05, ^**^P < 0.01, ^***^P < 0.001. 3V, 3rd ventricle; VLPO, ventrolateral preoptic nucleus.

In order to clarify whether the orexinergic axonal terminals affect sleep by releasing orexin on neurons containing orexin receptors in the VLPO of mice with conditioned fear, a microcannula was implanted into the VLPO of WT mice to apply orexin-A and electrodes were implanted to record EEG/EMG signals (Figures 7A, C). Compared to the controls, mice with conditioned fear got same changes in time in NREM, REM sleep and wakefulness as reported above (control vs. fear group: 42.06 ± 3.65% vs. 32.52 ± 2.29%, 6.7 ± 0.41% vs. 5.48 ± 0.51%, 51.24 ± 3.62% vs. 62.18 ± 2.27%, *t* = 4.944, 4.175, and 5.723, *P* = 0.001, 0.003, and 0.0004, *n* = 5 mice per group) (Figures 7D, F). After the injection of orexin-A into the VLPO, we found that the NREM and REM sleep time significantly decreased and wakefulness time significantly increased in mice with conditioned fear (fear vs. orexin-A group: 32.52 ± 2.29% vs. 28.1 ± 3.16%, 5.48 ± 0.51% vs. 4.14 ± 0.36%, 62.18 ± 2.27% vs. 67.76 ± 3.34%, *t* = 2.528, 4.829, and 3.091, *P* = 0.035, 0.001, and 0.015, *n* = 5 mice per group) (Figures 7D, F).

**FIGURE 8:**
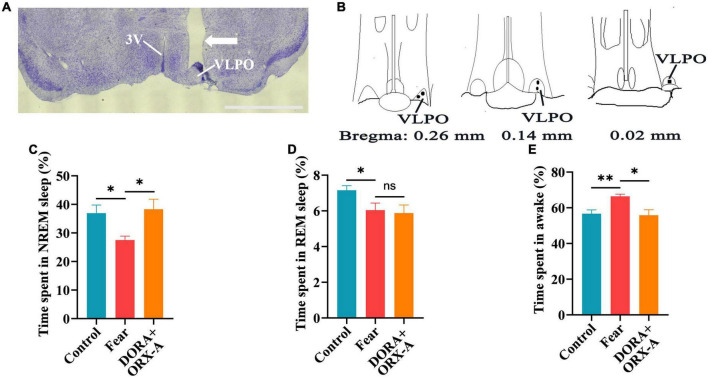
The effects of orexin-A in the VLPO were blocked by a pre-administrated orexin receptor antagonist (DORA). **(A)** The image of implantation site of cannula in the VLPO of mice. **(B)** The black spot representing the placement of the cannula in the VLPO of mice (*n* = 5 mice). **(C)** The comparison of the percentage of 24 h non-rapid eye movement (NREM) sleep time in control, conditioned fear and DORA + orexin-A mice (*n* = 5 mice per group). **(D)** The comparison of the percentage of 24 h rapid eye movement (REM) sleep time in control, conditioned fear, and DORA + orexin-A mice (*n* = 5 mice per group). **(E)** The comparison of the percentage of 24 h wakefulness time in control, conditioned fear, and DORA + orexin-A mice (*n* = 5 mice per group). Scar bar: **(A)** 1,000 μm. *P < 0.05, ^**^P < 0.01. DORA + ORX-A, DORA + orexin-A. 3V, 3rd ventricle; VLPO, ventrolateral preoptic nucleus.

We further determined if the effects of orexin-A in the VLPO were blocked by its antagonist. A microcannula was implanted into the VLPO of WT mice to apply orexin-A and its receptor antagonist, and EEG/EMG apparatuses were also installed to evaluate sleep (Figures 8A, B). Compared with the control mice, the NREM and REM sleep time significantly decreased and the wakefulness time significantly increased in mice with conditioned fear (control vs. fear group: 36.92 ± 6.35% vs. 27.54 ± 2.99%, 7.16 ± 0.60 vs. 6.04 ± 0.9%, 56.7 ± 4.82% vs. 66.42 ± 2.75%, *t* = 2.989, 2.392, and 3.917, *P* = 0.017, 0.044, and 0.004, *n* = 5 mice per group) (Figures 8C, E). When the dual orexin receptor antagonist (DORA) was pre-applied into VLPO 20 min before orexin-A applications in mice, a significant increase in the NREM sleep time and a decrease in the wakefulness time were observed in mice with conditioned fear (fear vs. DORA + ORX-A group: 27.54 ± 2.99% vs. 38.32 ± 7.78%, 66.42 ± 2.75% vs. 55.8 ± 7.0%, *t* = 2.894 and 3.160, *P* = 0.020 and 0.013, *n* = 5 mice per group) (Figures 8C, E).

## Discussion

In this study, the neural pathways from hypothalamic orexin neurons to the VLPO were demonstrated and an increased activation of these pathways was observed in mice with conditioned fear. Optogenetic activation of hypothalamic orexin neurons or hypothalamic orexin-VLPO neural pathways significantly decreased sleep time and increased wakefulness time. Similarly, the decreases in sleep time and increases in wakefulness time were occurred after the injection of orexin-A into VLPO and these effects of orexin-A in the VLPO were blocked by a pre-administrated orexin receptor antagonist.

Fear is a kind of stress response when the body suffer from danger or traumatic events (Fenster et al., 2018). Studies has shown that sleep impairments have significant effects on the generation and expression of fear, and fear also can cause sleep impairments, such as frequent nightmares, insomnia, and sleep fragmentation (Pace-Schott et al., 2022), but the mechanism by which fear causes sleep impairments is still unclear. The present study demonstrated that the neural pathways from hypothalamic orexin neurons to the VLPO were activated in mice with conditioned fear and sleep-wake changes were observed when the pathways were artificially regulated. These results suggest that hypothalamic orexin-VLPO neural pathways could mediate sleep impairments induced by conditioned fear.

Earlier studies found that the patients with narcolepsy lack orexin signaling, suggested that orexin neurons play an important role in maintaining arousal (Mahoney et al., 2019). The cell bodies of orexin neurons were localized exclusively in a subregion of the tuberal part of the hypothalamus and their axonal projections were widely distributed in the brain (Peyron et al., 1998), including the VLPO, a critical nucleus in sleep-wake regulation (Chou et al., 2002). Our present experiment verified that the orexin neurons projected to the VLPO by retrograde tracing technique combined with immunofluorescence staining in mice, this result is consistent with previous reports. Previous studies reported that orexin neurons are involved in modulating behavioral fear expression, and lack of orexin signals can lead to impaired fear response (Soya et al., 2013; Soya et al., 2017). Our results revealed that those VLPO-projected orexin neurons exhibited a high activation in mice with conditioned fear. When we further conducted an optogenetic activation of the hypothalamic orexin neurons or hypothalamic orexin-VLPO neural pathways in mice with conditioned fear, the aggravated sleep impairments were induced. These results strongly suggest that the hypothalamic orexin-VLPO neural pathways involved in fear-induced sleep impairments.

The hypothalamic peptides orexin-A interacts with orexin1 and orexin2 receptors to exert corresponding physiological functions (de Lecea et al., 1998; Sakurai et al., 1998). Histological studies have shown the high-level expressions of orexin1 and orexin2 receptors in the VLPO and they play a unique role in regulating wakefulness (Marcus et al., 2001). Orexin-A injected into the VLPO significantly increased wakefulness and reduced NREM sleep and REM sleep (Mavanji et al., 2015; Coborn et al., 2017). In this study, microinjection of the orexin-A into the VLPO of mice with conditioned fear resulted in an increased wakefulness and decreased sleep, suggesting that the orexin-A in the VLPO play a role in regulation of fear-related sleep impairments. The further experiment indicated that when the functions of orexin in the VLPO were blocked by local DORA, the sleep impairments in conditioned fear mice were reversed. These results suggest that the orexinergic axonal terminals in the VLPO played critical roles in regulating sleep-wake by releasing orexin and acting on VLPO neurons expressing orexin receptors in mice with conditioned fear.

Inconsistent with the expectation, photogenetic inhibition of the hypothalamic orexin-VLPO pathway did not change the sleep-wake in mice with conditioned fear. One possibility is that the wide orexinergic projections in the other brain regions, such as the cortex (Li et al., 2010), the locus coeruleus and dorsal raphe nucleus (Peyron et al., 1998; Mieda et al., 2011), and the paraventricular thalamus (Ren et al., 2018), give off a compensatory effect on wake or arousal when the orexinergic terminals in the VLPO were inhibited, but this needs additional experimental evidence. Another possibility is that NpHR3.0-mCherry is less efficient when it acts on presynaptic terminals (Bansal et al., 2020), therefore, more effective inhibition experiments are needed.

In summary, the present study has revealed that conditioned fear could induce the sleep impairments and further found that the hypothalamic orexinergic neurons and hypothalamic orexin-VLPO neural pathways were activated by conditioned fear. Additionally, the study has displayed that enhancement of the functions of these pathways by optogenetic activation or orexin-A microinjection can aggravate sleep impairments in mice with conditioned fear, and the sleep impairments in mice with conditioned fear can be reversed by local application of orexin receptor antagonist into the VLPO. These results strongly suggest that the neural pathways from hypothalamic orexinergic neurons to the VLPO are crucial pathways for sleep impairments induced by conditioned fear.

## Materials and methods

### Animals

Mice were placed at a temperature of 22 ± 1°C (40-60% humidity) and subjected to a 12 h light/dark cycle with free access to food and water. 48 C57/BL (wild type, WT) male mice (8–10 weeks of age) and 15 Hcrt-IRES-Cre male mice (8–10 weeks of age) were used in this experiment. WT mice were purchased from the Laboratory Animal Center of Ningxia Medical University. Hcrt-IRES-Cre mice were acquired by from Nanmo creatures. The study has been carried out in accordance with the governmental regulations on the use of experimental animals with prior approval by Laboratory Animal Ethical and Welfare Committee of Laboratory Animal Center of Ningxia Medical University (Document # IACUC-NYLAC-2021-152).

### General experiment protocol

Experiment 1 is to verify the conditioned fear induced sleep impairments: Part 1, the mice (*n* = 12) were tested freezing behavior of 24 h after the conditioned fear training and the percentage of freezing time was calculated. Part 2, the mice (*n* = 5) were implanted with EEG and EMG electrodes and were put back home-cages for 1 week to recover. Then, the mice were transferred to the recording chamber and connected with the recording line for 2 days followed by EEG and EMG recording for 24 h. After conditioned fear training, EEG and EMG were recorded again for 24 h. EEG and EMG signals were used to analyze the sleep-wakefulness states before and after conditioned fear training.

Experiment 2 is to identify the neural pathways from the hypothalamic orexin neurons to the VLPO: Mice (*n* = 5) were injected with CTB into the VLPO. One week later, the mice were sacrificed, and the coronal brain sections containing hypothalamus and VLPO were collected for immunofluorescence staining for CTB and orexin to identify the pathways from the hypothalamic orexin neurons to the VLPO.

Experiment 3 is to observe the activation of hypothalamic orexin-VLPO neural pathways in mice with conditioned fear: Part 1, four mice were received conditioned fear training (fear group), the other four mice were not received any training (control group). 1.5 h later, the mice were sacrificed and the coronal brain sections containing hypothalamus and VLPO were collected to observe activation of orexin neurons in hypothalamus by immunofluorescence staining for orexin and c-Fos. Part 2, injections of CTB into the VLPO were made in eight mice. One week later, four of those mice were received conditioned fear training (fear group), the left four mice were not received any training (control group). 1.5 h later, the mice were sacrificed and the coronal brain sections containing hypothalamus and VLPO were collected to observe activation of hypothalamic orexin-VLPO neural pathways by immunofluorescence staining for CTB, orexin and c-Fos.

Experiment 4 is to examine the change of sleep-wake induced by optogenetic activation or inhibition of the hypothalamic orexin-VLPO neural pathways in mice with conditioned fear: Part 1, Cre-dependent AAVs, AAV-DIO-ChR2-mCherry, were injected into hypothalamus of Hcrt-IRES-Cre mice (*n* = 5). Two weeks later, the mice were implanted with EEG and EMG electrodes, and an optical fiber was implanted into the hypothalamus. EEG and EMG were recorded for 24 h before and after conditioned fear training. Then, the blue optical activation was applied to orexin neurons in hypothalamus and EEG and EMG were recorded for 24 h (*n* = 5). Part 2, all of the operations were similar with that of Part 1, but the optical fiber was implanted into the VLPO for activation of orexinergic axonal terminals in the VLPO. Part 3, all of the operations were similar with that of Part 2, but the Cre-dependent AAVs that injected into hypothalamus is AAV-DIO-NpHR3.0-mCherry, and yellow optical stimulation were applied to the VLPO to inhibit the orexinergic axonal terminals in the VLPO (*n* = 5).

Experiment 5 is to test the function of orexin-A on hypothalamic orexin-VLPO pathways mediating sleep impairments induced by conditioned fear: Part 1, WT mice (*n* = 5) were implanted with EEG and EMG electrodes and an microcannula was implanted into the VLPO. EEG and EMG were recorded for 24 h before and after conditioned fear training. Then, orexin-A was applied into the VLPO through the microcannula and EEG and EMG were recorded for 24 h. Part 2, to determine if the effects of orexin-A in the VLPO could be blocked by a pre-administrated orexin receptor antagonist, the other five WT mice were used to conduct similar protocols as Part 1, but a DORA application was made into the VLPO 20 min prior to orexin-A application (*n* = 5).

### Behavioral experiment

In Experiment 1, the mice were put into the experimental room 1 h in advance on the day of experiment. The mice were placed in the shuttle box (MED-APA-D1R, Zenda Limited Liability Company, Shanghai, China) for 2 min (adaptation period), they were given sound stimulation (75 dB, 2,900 Hz) for 30 s, followed by unavoidable plantar electric shock (0.6 mA, lasting 2 s) for the next 2 s. The sound-electric shock was paired for five times with an interval of 2 min each time (Soya et al., 2013).

Test sessions were performed in 24 h after the conditioned fear training, the mice were placed in the training environment without any stimulation. The freezing time was recorded within 5 min, and the percentage of freezing time was calculated. The percentage of freezing time (%) = freezing time (s)/total time (s) × 100%. The behavior of freezing is the way of fear expression in the rodent, which is manifested by the loss of all muscle movements except breathing. The percentage of freezing time reached 30% indicates the successful preparation of conditioned fear model.

### Stereotaxic injections of retrograde tracer CTB and virus

For microinjection of CTB (Experiment 2 and 3), WT mice were anesthetized with isoflurane (1.0–1.5%, RWD Life Science, Shenzhen, China) and then were fixed on a stereotaxic apparatus (RWD Life Science, Shenzhen, China) with the skull plane in the horizontal position. The hair on head were shaved and a longitudinal cut was made on the skin to expose the cranium. The bregma was used as the reference point for injection. A 1–2 mm hole was drilled on the cranial bone and the micro-syringe filled with CTB (0.2%, 30 nl, Lot#10433A1, List Labs, USA) was gradually injected into the unilateral VLPO (bregma: *AP* = + 0.26 mm, *ML* = –0.6 mm, *DV* = –5.5 mm) of mice.

For microinjection of virus (Experiment 4), Hcrt-IRES-Cre mice were anesthetized and operated as CTB injection mentioned above. The AAV-DIO-ChR2-mCherry [rAAV-EF1a-DIO-hChR2 (H134R) -mCherry-WPRE-hGH pA, AAV2/9, 1.5 × 10^13^ virus molecules/ml; provided by Brain VTA, Wuhan, China] or AAV-DIO-NpHR3.0-mCherry (rAAV-EF1a-DIO-eNpHR3.0-mCherry-WPRE-pA, AAV2/9, 1.5 × 10^13^ virus molecules/ml; provided by Brain VTA, Wuhan, China) was injected into the bilateral hypothalamus of mice (200 nl on each side, bregma: *AP* = –1.5 mm, *ML* = ± 0.7 mm, *DV* = –5.3 mm). Two weeks later, mice underwent a second surgery to implant EEG and EMG and optical fiber.

### Implantation of EEG/EMG electrodes, optical fiber, and microcannula

Mice were anesthetized with isoflurane and fixed on the stereotaxic apparatus, and then the skull was exposed. Two stainless steel screws were fixed to the anterior frontal bone and another screw was fixed to the posterior cerebellar skull region. Two EMG electrodes were also inserted into the neck muscles. Insulated leads of EEG and EMG electrodes were soldered to screw. For Experiment 4, the optical fiber (200 μm, internal diameter, RWD, Cat. R-FOC-L200C-50NA) was implanted into the hypothalamus (Bregma: *AP* = –1.5 mm, *ML* = ± 0.7 mm, *DV* = –5.0 mm) or VLPO (Bregma: *AP* = + 0.26 mm, *ML* = ± 0.6 mm, *DV* = –5.3 mm) of mice. For Experiment 5, the stainless microcannula (Cat#62102, RWD Life Science, Shenzhen, China) was implanted into the VLPO of mice. Finally, dental cement was applied to completely cover the exposed skull and devices implanted. Mice were allowed to recover in their home-cages for 1 week.

### Drugs

Orexin-A (15.6–125 pmol/0.5 μl, Cat# 003-30, Lot# 43320, PHOENIX PHARMACEUTICALS, INC, Shanghai, China) was dissolved in artificial cerebrospinal fluid for the injection studies with orexin-A. DORA (TCS-1102, 62.5–250 pmol/0.5 μl, Catalog. No: 3818, TOCRIS bio-techne) were dissolved in DMSO/methanol HCl/sterile water. All drugs were stored at 4°C for < 48 h.

### EEG/EMG recording, optogenetic stimulation, and drugs injection

In Experiment 1, the mice were transferred to the recording chamber and connected to the recording line for 2 days followed by EEG and EMG recording for 24 h. After conditioned fear training, EEG and EMG were recorded for 24 h. In Experiment 4, the mice were transferred to the recording chamber and connected to the recording line for 2 days followed by EEG and EMG recording for 24 h. After conditioned fear training, EEG and EMG were recorded for 24 h. Finally, optical activation (10 Hz, 10 ms per pulse, 15 mW energy, 473 nm blue light, lasting for 120 s, PlexBright, Neurotechnology Research Systems) or inhibition (10 Hz, 10 ms per pulse, 15 mW energy, 590 nm yellow light, lasting for 120 s) were conducted and the EEG and EMG were recorded for 24 h. Optical activation were performed during 9:00–17:00 while optical inhibition were performed during 21:00–5:00. A refractory period of at least 20 min was allowed between each stimulation. Each mouse received five stimulations in recording process. In Experiment 5, part 1, mice were transferred to the recording chamber and connected to the recording line for 2 days followed by EEG and EMG recording for 24 h. After conditioned fear training, EEG and EMG were recorded for 24 h. Finally, orexin-A (15.6–125 pmol/0.5 μl) was gradually injected with a 30-gauge injector-single (Cat#62203, RWD Life Science, Shenzhen, China) into the VLPO in mice with conditioned fear, EEG and EMG were recorded for 24 h; Part 2, the mice were transferred to the recording chamber and connected to the recording line for 2 days followed by EEG and EMG recording for 24 h. After conditioned fear training, EEG and EMG were recorded for 24 h. Finally, DORA (62.5–250 pmol/0.5 μl) was injected into the VLPO 20 min prior to orexin-A (15.6–125 pmol/0.5 μl), and EEG and EMG were recorded for 24 h. Injections were performed between 8:00 to 10:00.

### Brain tissue preparation, immunofluorescence, and Nissl staining

Mice were deeply anesthetized with isoflurane and transcardially perfused with 200 ml of 0.9% saline, followed by 50 ml of 4% paraformaldehyde. Brain tissues were post-fixed with 4% paraformaldehyde (8 h), dehydrated with 30% sucrose (48 h) and then cut into 40 μm coronal brain tissue sections using a flat-push freezing microtome (Leica, Germany).

In Experiment 2, double immunofluorescence staining for CTB and orexin was conducted to observe the CTB injection site and retrograde labeling of hypothalamic orexin neurons. Brain sections containing the hypothalamus and VLPO were incubated in goat anti-CTB (1:1000, Lot# 7032A9, List Labs, USA) and rabbit anti-orexin-A (1:1000, Abcam, USA) primary antibodies overnight at 4°C. Then, sections were washed in PBS three times, followed by incubation with Cy™3-conjugated polyclonal donkey anti-goat IgG (1:500, Lot# 134527, Abcam, USA) and Alexa fluor^®^488-conjugated polyclonal donkey anti-rabbit IgG (1:500, Lot# 136422, Abcam, USA) secondary antibodies for 3 h at room temperature. Sections were washed three times in PBS, mounted on slides, coverslipped using fluorescent sealant (Beijing Zhongshan Jinqiao Biotechnology Co. LTD).

In Experiment 3, double immunofluorescence staining for orexin and c-Fos was conducted to study the possible activation of hypothalamic orexin neurons after conditioned fear. Brain tissue sections of containing the hypothalamus were incubated in rabbit anti-orexin-A (1:1000, Abcam, USA) and rat anti-c-Fos (1:1000, Cat# 226017, Synaptic Systems, USA) primary antibodies overnight at 4°C. Then, sections were washed in PBS three times, followed by incubation with Alexa fluor^®^488-conjugated polyclonal donkey anti-rabbit IgG (1:500, Lot# 136422, Abcam, USA) and Cy™5-conjugated polyclonal donkey anti-rat IgG (1:500, Lot#154628, Abcam, USA) secondary antibodies for 3 h at room temperature. Sections were washed three times in PBS, mounted on slides, coverslipped using fluorescent sealant and observed under the LAS X microscope. Triple immunofluorescence staining for CTB, orexin and c-Fos was conducted to study that the hypothalamic orexin-VLPO neural pathways were activated by conditioned fear. Brain tissue sections containing the hypothalamus and VLPO were incubated in Goat anti-CTB (1:1000, Lot# 7032A9, List Labs, USA), rabbit anti-orexin-A (1:1000, obtained by the Abcam, USA) and rat anti-c-Fos (1:1000, Cat# 226017, Synaptic Systems, USA) primary antibodies overnight at 4°C. Then, sections were washed with PBS three times, followed by incubation with Cy™3-conjugated polyclonal donkey anti-Goat IgG (1:500, Lot#134527, Abcam, USA), Alexa fluor^®^488-conjugated polyclonal donkey anti-Rabbit IgG (1:500, Lot#136422, Abcam, USA) and Cy™5-conjugated polyclonal donkey anti-rat IgG (1:500, Lot#154628, Abcam, USA) secondary antibodies for 3 h at room temperature. Brain tissue sections were washed three times in PBS, mounted on slides, coverslipped using fluorescent sealant.

In Experiment 4, double immunofluorescence staining for orexin and ChR2-mCherry or NpHR3.0-mCherry was conducted to verify the selective expression of ChR2-mCherry or NpHR3.0-mCherry in orexin neurons. Brain tissue sections containing the hypothalamus were incubated in rabbit monoclonal anti-orexin-A (1:1000, Abcam, USA) primary antibodies overnight at 4°C. Then, brain tissue sections were washed in PBS three times, followed by incubation with Alexa fluor^®^488-conjugated polyclonal donkey anti-rabbit IgG (1:500, Lot#136422, Abcam, USA) secondary antibodies for 3 h at room temperature. Sections were washed three times in PBS, mounted on slides, coverslipped using fluorescent sealant.

In Experiment 5, Nissl staining was used to observe the position of microcannula implantation. Brain sections were mounted on slides and air dried. Sections were placed in Nissl staining solution A (Beijing Solarbio science & technology CO., Ltd.) at 56°C for 1 h, followed by placed in 1% hydrochloric acid alcohol differentiation solution (100 ml differentiation solution contained 1 ml of 36% hydrochloric acid and 99 ml of 75% alcohol) for 2 min. Sections were placed in gradient alcohol 75, 85, 95, 100 I, and 100% II to dehydrate, and then were put into xylene I and xylene II in turn for 1 min. Finally, brain sections were coverslipped using neutral balsam (Beijing Solarbio science & technology CO., Ltd).

The sections were observed under the LAS X microscope (Leica, DM6B, Germany).

### EEG/EMG analysis, data quantification, and statistics

An 8-channel small animal system (Sleep Sign 3.0 for 8 Animals, Kissei Comtec, Japan) were used to record and analyze the EEG and EMG in mice. System automatically determined the recorded polymorphic signal as “WAKE,” “NREM” or “REM” state, the sampling rate of EEG and EMG signals was 256 Hz. The defined sleep-wake state was checked and corrected manually if necessary. Waveform characteristics of EEG and EMG in sleep-wake state: wakefulness was defined as an asynchronous, low-amplitude EEG rhythm and an increase in EMG activity accompanied by phase bursts, main theta waves (4–8 Hz). NREM sleep refereed to the brain electrical activity dominated by synchronous, high-amplitude, low-frequency (0.5–4 Hz) δ waves. Compared with waking state accompanied by phase bursts, NREM sleep had lower EMG activity. REM sleep was defined as having a distinct θ (4–10 Hz) rhythm with little or no EMG activity.

In Experiment 2, two mice with inaccurate injection sites for CTB were excluded from quantitative analysis, and the number of CTB labeled cell bodies and orexin^+^ colocalized cells in each section of hypothalamus were counted. Eight representative brain sections from each mouse were counted. In Experiment 3, the number of orexin^+^ and c-Fos^+^ colocalized cells in hypothalamus were counted. Three representative brain sections of each mouse were counted; and the number of CTB labeled cell bodies, orexin^+^ and c-Fos^+^ colocalized cells in hypothalamus were counted. Three representative brain sections of each mouse were counted. In Experiment 4, the number of mCherry^+^ and orexin^+^ colocalized cells in each section of hypothalamus were counted. Eight representative brain sections from each mouse were counted. The counting was performed manually by using the ImageJ counting tool.

Graph Pad Prism8.0.1 was used for data statistical. Numerical data are reported as the mean ± SEM. Non-paired *t*-test was used to calculate the *P*-values. Statistical significance was set at *P* < 0.05.

## Data availability statement

The raw data supporting the conclusions of this article will be made available by the authors, without undue reservation.

## Ethics statement

This animal study was reviewed and approved by Laboratory Animal Ethical and Welfare Committee of Laboratory Animal Center of Ningxia Medical University.

## Author contributions

JGN and ZYH conceived and designed the experiments, interpreted the data, and critically revised the manuscript. CM, NZ, KM, JDN, and TM performed the experiments and acquired the data. ZQH, YW, and CL analyzed the data. CM and NZ drafted the manuscript. All authors read and approved the final version of the manuscript.
